# Construction and Evaluation of a Preoperative Prediction Model for Lymph Node Metastasis of cIA Lung Adenocarcinoma Using Random Forest

**DOI:** 10.1155/2022/4008113

**Published:** 2022-09-25

**Authors:** Chuhan Zhang, Shun Xu, Youhong Jiang, Changrui Jiang, Shangxin Li, Zhitong Wang, Yan Dong, Feng Jin, Dan Zhao, Yating Zhao

**Affiliations:** ^1^Department of Thoracic Surgery, The First Hospital of China Medical University, Shenyang 10001, China; ^2^Cancer Institute, The First Hospital of China Medical University, Shenyang 10001, China; ^3^Department of Anesthesiology, The First Hospital of China Medical University, Shenyang 10001, China; ^4^Department of Public Health, The First Hospital of China Medical University, Shenyang 10001, China; ^5^Department of Neurology, The First Hospital of China Medical University, Shenyang 10001, China

## Abstract

**Background:**

Lymph node metastasis (LNM) is the main route of metastasis in lung adenocarcinoma (LA), and preoperative prediction of LNM in early LA is key for accurate medical treatment. We aimed to establish a preoperative prediction model of LNM of early LA through clinical data mining to reduce unnecessary lymph node dissection, reduce surgical injury, and shorten the operation time.

**Methods:**

We retrospectively collected imaging data and clinical features of 1121 patients with early LA who underwent video-assisted thoracic surgery at the First Hospital of China Medical University from 2004 to 2021. Logistic regression analysis was used to select variables and establish the preoperative diagnosis model using random forest classifier (RFC). The prediction results from the test set were used to evaluate the prediction performance of the model.

**Results:**

Combining the results of logistic analysis and practical clinical application experience, nine clinical features were included. In the random forest classifier model, when the number of nodes was three and the *n*-tree value is 500, we obtained the best prediction model (accuracy = 0.9769), with a positive prediction rate of 90% and a negative prediction rate of 98.69%.

**Conclusion:**

We established a preoperative prediction model for LNM of early LA using a machine learning random forest method combined with clinical and imaging features. More excellent predictors may be obtained by refining imaging features.

## 1. Introduction

Lung cancer is a malignant tumor with high morbidity and mortality rates. The latest global cancer data released by the International Agency for Research on Cancer (IARC) of the World Health Organization shows that the incidence of lung cancer ranks second and mortality ranks first among all cancers, and the morbidity and mortality rates rank first among cancers in China. Non-small-cell lung cancer (NSCLC) is the most common pathological type of lung cancer, accounting for 80% of all lung cancers [1].

Lymph node metastasis (LNM) is an important route of metastasis in lung cancer and the main factor affecting staging and prognosis. In recent years, with improvements in radiological techniques and increased frequency of regular physical examinations, the proportion of patients identified with early-stage NSCLC has increased. Additionally, because of the COVID-19 pandemic, the use of lung CT has increased [2, 3]. Increased application of lung CT improves the detection rate of early lung cancer.

While mediastinoscopy or PET is the gold standard for examining LNM in lung cancer [4, 5], these examinations are invasive and cause an economic burden to patients [5, 6]. Additionally, the diagnostic effect of PET on LNM of early NSCLC is not ideal [5, 7]. However, performing deep lymph node dissection for all early LA patients is invasive and not needed for all patients. Therefore, preoperative prediction of LNM in early LA is critical to identify patients that require surgery.

Previous studies have used logistic regression to construct prediction models for LNM, but the results of the models tended to explain only the importance and application of risk factors [8–11]. The predictive ability of the models for LNM is not clear, and the prediction results of LNM-positive cases remain unsatisfactory. With the increasing applications of artificial intelligence, ML has gradually become a hot research area for building prediction models. Research has shown that the prediction efficiency of the ML model is better than that of the traditional linear regression model [12]. Therefore, the purpose of this study was to establish a suitable preoperative prediction model of LNM in early LA by summarizing imaging findings and clinical features of early NSCLC, combined with statistical methods and ML. This model will help reduce unnecessary lymph node dissection and surgical injury and shorten surgical time.

## 2. Materials and Methods

### 2.1. Selection of Cases

**Figure 1:**
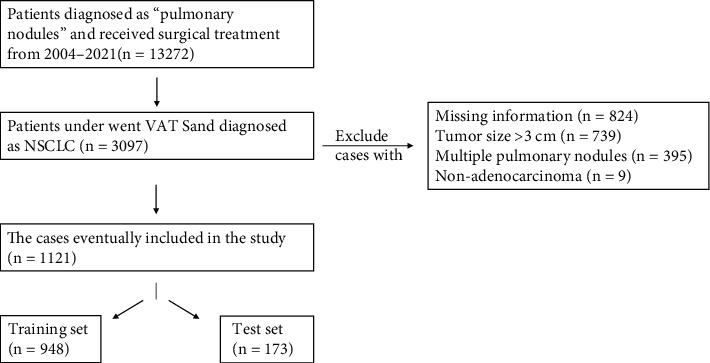
Flowchart of patient selection and exclusion.

We retrospectively reviewed 13272 patients with lung tumors in the Department of Thoracic Surgery of the First Hospital of China Medical University between January 2004 and October 2021. We preliminarily selected 3097 patients who underwent VATS and were diagnosed with NSCLC. Patients with incomplete data and non-cIA or multiple tumors were excluded, and 9 of the remaining 1130 patients were excluded because intraoperative frozen sections were later confirmed as nonadenocarcinoma.  Figure 1 shows the patient selection process. This study was approved by the Institutional Ethics of Committee of the First Hospital of China Medical University (2021-440).

### 2.2. Clinicopathological Variables

All 1121 enrolled patients with early solitary LA (≤3 cm) underwent VATS resection and lymph node dissection at the First Hospital of China Medical University. All clinicopathological information was collected in the hospital information system (HIS), with CT images (thin layer, 1.25 mm and under) and pathological results. All cases had received lung CT results within one month before operation. Two thoracic surgeons reread the CT images of the patient group to measure nodule characteristics and restage the lung cancer following the eighth edition of TNM staging of lung cancer. In cases of disagreement, a radiologist determined the final conclusion.

The average number of lymph node dissections was 9. A total of 64 cases were confirmed with LNM by postoperative pathology, including 20 cases with masses of 2 cm or smaller and 44 cases with masses larger than 2 cm. There were 17 cases of N1a, 13 cases of N1b, 21 cases of N2a, and 13 cases of N2b.

Following the results of previous similar studies [8–12] and our clinical experience, we preliminarily selected 20 features for this study, including tumor location (lobe/zone), vascular shadow, pleural indentation, lobulated and spiculated sign, average long- and short-axis diameters of the solid components (mediastinal window/lung window), solid area of the tumor (mediastinal window/lung window), maximum area of the tumor (lung window), consolidation tumor ratio (mediastinal window/lung window), maximum tumor diameter (lung window), age, sex, enlargement of lymph nodes (ELN), PaO_2_, PaCO_2_, CEA, and NSE.

### 2.3. Univariate Analysis

Univariate analysis was performed using IBM SPSS (version 25.0; SPSS, Inc., Chicago, IL, USA) to screen the influencing factors. Univariate logistic regression was selected for data analysis using postoperative lymph node pathology as the variable.

A *P* value < 0.05 was considered statistically significant. Continuous variables are expressed as mean ± standard deviation (SD), and categorical variables are described with frequencies. For similar variables, we used the ROC curve (from 1121 cases) to measure the work efficiency and identify suitable variables.

### 2.4. Construction of RFC Model

The random forest algorithm was used to build the prediction model using the R programming language (version 4.1.2). We included 173 cases (including 20 LNM-positive cases) from the total sample into the test set, and the remaining 948 cases (including 44 LNM-positive cases) were included in the training set. The prediction ability of the model was verified with real cases, and the verification results were subject to postoperative pathology.

## 3. Results

**Figure 2:**
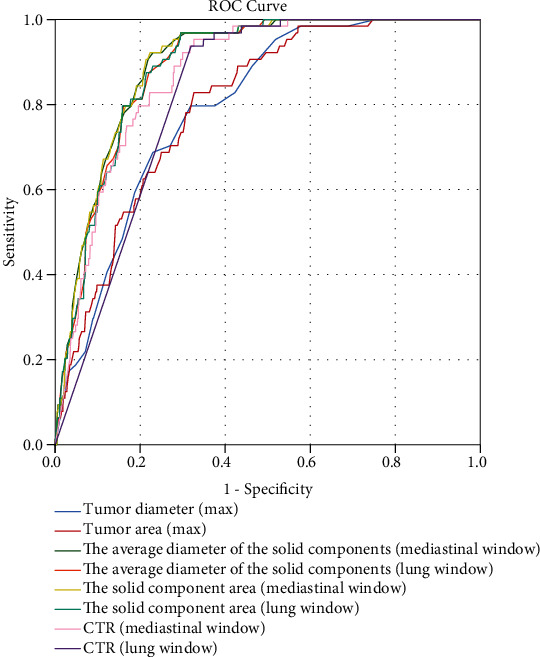
ROC curve of similar variables.

Table 1 shows the variables included in the study and univariate logistic analysis results. As shown in Figure 2 and Table 2, we used the mediastinal window consolidation tumor ratio (AUC = 0.873) as the final CTR. We also chose the solid area with a mediastinal window (AUC = 0.896) as the model variable to reduce the impact of cases with the same CTR but different tumor sizes.

In the variable selection, we unexpectedly found that the PaCO_2_ was significantly associated with LNM (*P* < 0.05). However, we had no way to confirm a relationship between this variable and LNM, and therefore, it was not included in the final ML model.

**Figure 3:**
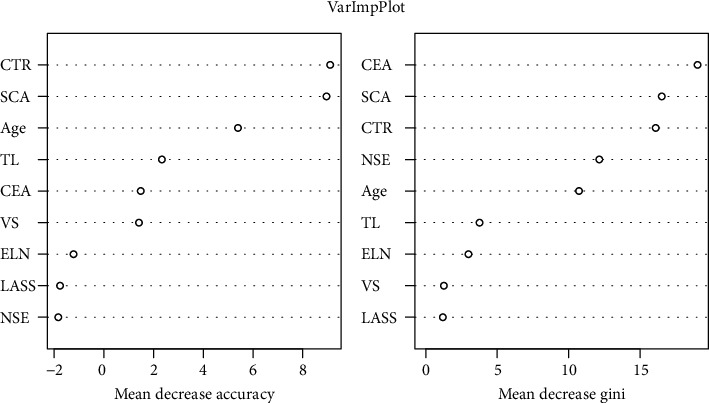
Importance and stability of each variable.

On the basis of our clinical experience and the univariate logistic analysis results, nine variables were selected for inclusion in the final ML steps. When *n*‐tree = 500 and the number of classification nodes was three, the model achieved the best performance. On this basis, we compared the probability given by the model and adjusted its cut-off point. The test results showed that the positive prediction rate of the model was 90%, the negative prediction rate was 98.69%, and the accuracy rate was 97.69%.  Table 3 presents the evaluation indices of the RFC model, and Figure 3 shows the importance and stability of these variables.

Using this model, we monitored 100 patients with solitary LA (≤3 cm) in the First Hospital of China Medical University from February to May 2022. During this period, five patients with isolated 2–3 cm LA had a probability for LNM positivity of more than 10%, and two of these patients were diagnosed as LNM-positive by the model. Mediastinal and intrapulmonary lymph nodes were carefully examined after operation. The results indicated that two cases with positive predicted results showed N1 and N2 metastasis. Among the three cases with negative predicted results but a positive probability over 10%, two cases were N1 and one case had no metastasis. The results were similar to those of our tests.

We calculated the cut-off values of the first two continuous variables in the ranking given by the model. Using Youden's index as the standard, the solid area measured with mediastinal window greater than 1.55 (59/64, 297/1121) and CTR higher than 45.2% (61/64, 406/1121) would significantly increase the probability of LNM. These may provide some data basis for further clinical study of lymph node metastasis.

## 4. Discussion

In recent years, with improvements in radiological techniques and increased frequency of regular physical examinations, the proportion of patients with early-stage NSCLC has increased. Owing to the low metastasis rate and small tumor size, the methods of early-stage NSCLC resection and lymph node dissection are constantly being updated and improved by surgeons worldwide, to promote the development of surgical precision medicine.

The treatment of NSCLC, especially LA, has been a major focus of research. Many studies have explored the identification of meaningful prognostic factors and new treatments [13–16]. However, improving methods for early detection of LNM not only helps determine whether patients should undergo further examination but also has great guiding significance for lymph node dissection during surgery. At present, tumor size, CTR, tumor markers, and imaging features have been repeatedly confirmed as preoperative predictors of LNM in lung cancer [17–28]. In clinical treatment, biopsy is the gold standard to determine the status of LNM. However, biopsy is an invasive examination and therefore, establishing a prediction model of LNM for prebiopsy use is important. Some studies have indicated that PET can be used for preoperative observation, with 3.3 identified as the cut-off value of SUVmax [29]. However, other studies have reported that PET has no significant effect on observing LNM of early NSCLC [30–35]. Therefore, it is not advisable to use PET in clinical treatment to observe the presence of LNM in early small nodules. In our database in this study, patients with early pulmonary nodules who underwent preoperative examination with PET accounted for 6.8% of the total patient group, and only 11.7% of all metastatic cases underwent PET before surgery, which indicates that a large number of patients with LNM requiring PET examination have not been accurately identified, even including some patients whose tumor size was less than 2 cm. Therefore, establishing a predictive model that can accurately predict LNM before surgery is an important and challenging task.

To establish an accurate clinical prediction model, we first performed strict selection and measurement of variables. Several studies have confirmed that CTR, the ratio of the solid component diameter to the maximum tumor diameter, is closely related to LNM [24–26]. While some software can measure the tumor volume ratio, they are not widely used, and thus, CTR remains the first choice for many clinicians. However, most studies on the tumor consolidation rate only measured the ratio of the two length diameters, without considering the short diameter. In 2017, the Fleischner Society published guidelines on CT imaging identification of pulmonary nodules [36], which proposed that the measurement of solid components should include both long and short axes.

**Figure 4:**
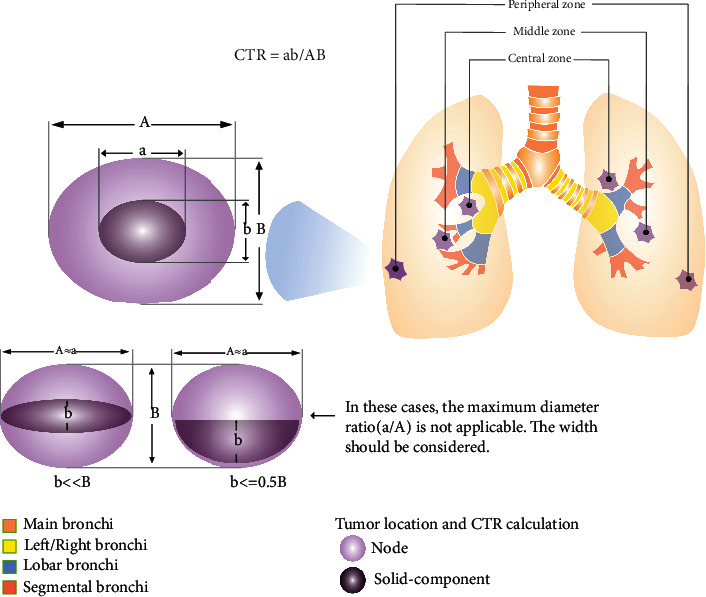
Tumor location and CTR calculation.

As shown in Figure 4, when the maximum diameter of the solid component is close to the maximum diameter of the solid component, the key point of CTR is the ratio of the width, especially for tumors in which the width of the solid component is much smaller than the maximum width of the tumor. Even if the length ratio is 1, it does not mean that these tumors are pure solid tumors. Therefore, we changed the CTR from the length ratio to the area ratio to avoid the influence of large differences between the length and width of the tumor. This is one of the main differences between our model and the previous prediction models that included CTR.

We also propose a new classification method for tumor location in CT images using the location relationship between the tumor and the segmental bronchus. When there was an observable segmental bronchial shadow around the tumor, we designated the tumor location in the middle zone; tumors located above the segmental bronchus, with unclear boundaries from the mediastinum or lobar bronchus, were considered to be located in the inner zone, and tumors located below the segmental bronchus, without bronchial shadow, were located in the outer zone. This approach describes the location of the tumor more precisely than other descriptions of central and peripheral types.

We also compared the same continuous variable with different windows. The AUC results showed that the measurement results of solid components with a mediastinal window are more suitable for the calculation of CTR. Our study also showed that variables that include both long and short diameter of the tumor are better than those only including the long diameter. However, there was no significant difference between the area of solid components and average long- and short-axis diameters of the solid components in the ROC results. To facilitate the application and calculation of clinical treatments, we believe that the average diameter of the solid components can be used directly.

Several models have been reported for predicting LNM of NSCLC, and most of these are logistic regression models [8–11]. However, most models showed high specificity and low sensitivity, which indicates that they are unable to distinguish between true-negative cases and true-positive cases. With the increasing application of artificial intelligence (AI), ML has gradually become a widely used option for building prediction models. Wu et al. [12] summarized the commonly used prediction models and compared their prediction ability. The results showed that the prediction efficiency of the ML model is significantly better than that of the traditional multifactor model, and the RFC model performed better in the prediction of preoperative LNM.

In this study, we first attempted to build a prediction model using a traditional logistic multifactor regression analysis. The results were similar to those of many previous studies [8–11], and the negative predictive value of the model was very high. However, it was difficult to achieve the desired positive predictive value; even when we changed the cut-off point to 0.1, the sensitivity did not reach 0.8 and more than 100 negative patients were predicted to be positive cases. Therefore, logistic multifactor regression analysis may not be a suitable method for preoperative prediction of LNM.

We then used ML and R programming to build the RFC model. We compared the computational probability of positive and negative cases in the internal test set constructed by RFC from the training set data. After comparison, we found a group difference in the probability of positive and negative cases calculated by the model. Most of the positive cases had a positive prediction probability of more than 20%, whereas the negative cases had a positive prediction probability of markedly less than 10% or even lower than 1%. Therefore, we set the cut-off point of the model; when a case was calculated to have a 20% probability of metastasis, the model classified it as a positive case.

The results of the new model are satisfactory. After retesting, the accuracy of the model was 97.69%, the positive prediction rate was 90%, and the negative prediction rate was 98.69%. Even if the cut-off value is low (20%), the false-positive rate of the model is still less than 2%, which shows that the model is very effective for the classification of test set cases. We were able to completely screen out all true-negative cases and accurately identify the few positive cases. The meta-analysis by Birim et al. [37] showed that the overall sensitivity and specificity rates of PET in the detection of mediastinal LNM were estimated to be 83% and 92%, respectively. Compared with the existing prediction model research and the results of PET, our model performed better.

Compared with previous prediction models of the same type, our model has a larger data volume and a more refined data collection in that it included tumors in all locations rather than only tumors in the peripheral location [12, 38, 39]. Similar to Wu et al. [12], we did not exclude pure GGO and GGO-dominant part-solid tumors in this model construction, because the CTR in this study was different from CTR in other studies. In our data, five of the patients with CTR < 0.5 had lymph node metastasis, which was the main reason why we did not exclude pure GGO and GGO-dominant part-solid tumors. Additionally, more sufficient and complete data means a more efficient model.

We also generated statistics on the prediction results of the LNM at N1 station. Among the six cases of N1 metastasis in the test set, four cases were accurately predicted and two cases showed false-negative results, which affected the positive predictive value. Because there were still false-negative cases and such errors cannot be easily ignored in clinical treatment, we compared the probability of cases in the test set given by the model (Table 4). The results were consistent with what we saw in our internal test set.

We also reviewed the data for false-positive cases in detail. The tumors were solid-dominant part-solid nodes; both of their maximum tumor diameters were over 1.8 cm, with pleural indentation and spiculated signs, and analysis of the intraoperative frozen sections revealed adenocarcinoma. Our clinicians performed complete systematic lymph node dissection during the operation, and no evidence of LNM was found. All characteristics of the case are in line with our current criteria for systematic lymph node dissection, and the prediction probability of the model was consistent with our actual treatment of the case. We believe that this may be a special case, but this case also confirms the homogeneity of the ML model and clinician thinking, to some extent. The results of the model were consistent with the actual treatment of the case.

The RFC model also gave the order of importance and stability of the variables introduced by the model (Figure 3). Variables related to tumor solid components and tumor size ranked very high, and the tumor solid area (mediastinal window) and CTR were the most prominent. However, ELN, as in our commonly used clinical observation, was not a key variable in this model. Only 28 of the 188 patients with mediastinal ELN had LNM, which accounted for only 43.5% (28/64) of all metastasis-positive cases. More than half of the patients with LNM did not show enlarged lymph nodes. We speculate that in these early-stage patients, the enlarged lymph nodes without metastasis are more likely caused by inflammation or hyperplasia. The ML model is entirely based on the training set data, which may be one of the main reasons for the poor performance of this variable.

Moreover, the sensitivity of tumor markers such as CEA and NSE may not be very high in early NSCLC. From our data review results, only 39% (25/64) of the total cases of metastasis had CEA greater than 4.30, whereas more than 93% of the cases with NSE greater than 16.30 had no LNM. These two indicators do not show much advantage in early prediction; therefore, they rank lower in importance descriptions. This may be because in patients with early LA, the effect of the tumor on the body is small, and the commonly used cut-off values of tumor markers are not applicable to this group. For patients with early LA, lower cut-off values may be more effective in identifying cases with high risk of metastasis. The LASS shows no advantage in the rank of importance, which is consistent with the results of previous studies [12].

We propose the following method for lymph node dissection in patients with isolated LA before surgery. When the prediction result of the model determines that a patient has metastasis, we choose systematic lymph node dissection; when the case is determined to be without metastasis, for patients with a metastasis probability of 10%–20%, more samples and more detailed pathological examination of the pulmonary lymph nodes are required. For patients with a positive probability of less than 10%, lobe-specific lymph node dissection and segmental pneumonectomy or wedge resection may be options. Patients with a positive probability of less than 1% can choose to undergo lymph node sampling and wedge resection. This strategy needs to be confirmed in clinical practice.

Using this approach, we randomly monitored 100 patients and 5 patients had a positive probability of more than 10%, including two patients who were determined to have LNM. Mediastinal and intrapulmonary lymph node examination showed that two cases with positive predicted results had N1 and N2 metastasis. Among three cases with negative predicted results but positive probability over 10%, two cases were N1 and one case had no metastasis. The calculated results of the other cases were less than 10%, and the pathological results also suggested that there was no LNM. The results were similar to those of our tests.

The test results show that the model can help clinicians predict the probability of LNM in patients with early lung adenocarcinoma before operation and further guide the scope of lymph node dissection during the operation.

Intraoperative pathological results should be combined with clinical experience; even if various indicators point to a high risk of metastasis, some solid nodules are tuberculosis or benign hamartoma. Pathological typing is not included as a model variable, and thus, the application of this model is not limited to the choice of intraoperative methods but it also helps determine which patients need more attention before operation, such as those with a positive probability of 10%–20%, who are more likely to have N1 metastasis rather than N2 metastasis, because this is the mean area in the model that cannot be accurately classified.

Our findings indicate that the group with LNM among patients with early isolated LA showed certain characteristics. In our study, the group with a positive probability of more than 20% was likely to have LNM. Mediastinal LNM is not common in patients with a positive probability of 10%–20%; most metastasis is N1 stage LNM. Although there is little difference in the predicted probability of LNM in these cases, we were able to distinguish them from patients without LNM (positive probability less than 1%). This may be a special advantage of ML models in producing better classification results by comparing subtle differences in data.

This study had several limitations. First, this was a single-center retrospective study and we used the same database for training and testing; however, we used some new variables as predictive variables, such as CTR from area ratio, and these variables cannot be found in the public database, which makes it impossible for us to test the model through external verification. Second, because of the clinical characteristics of early LA, there was a small proportion of cases with metastasis, leading to a lack of positive materials for ML. This was one of the main reasons for the difficulty in improving the sensitivity of the predictive model. Additionally, to build the model, we did not distinguish between N1 and N2 metastases; N1 probability was markedly lower than that of N2 (gap of approximately 10%–20%), which makes it necessary to adjust the cut-off point to obtain better results.

## 5. Conclusion

Our study was aimed at constructing a prediction model for preoperative LNM through ML to provide a strategy for reducing unnecessary surgical trauma and shortening the operation time. Using the random forest algorithm, we successfully built a prediction model; in the 173 patients in the test set, the model correctly predicted 18 cases of patients with LNM and 151 negative cases. From the specific probability calculated by the model, we were able to further distinguish the mispredicted cases from true-negative results. This was confirmed in subsequent verification of real cases.

The tumor solid component area and CTR were identified as the main predictive factors, whereas CEA and NSE were not sensitive to the prediction of early LA metastasis. Our RFC model reflected this phenomenon. Third, in the measurement and calculation of the solid components, the variables including both the long diameter and short diameter performed better than those with only the long diameter, and the results obtained under the mediastinal window performed better. From these variables, our ML model also shows great potential for development, which could help clinicians make lymph node dissection plans. This study is a good test for the preoperative prediction of LNM; it can provide more sufficient clinical basis for future research in this field.

## Figures and Tables

**Table 1:** Univariate analysis results of patients' variables (*n* = 1121).

Variable	Total	*P* value
Enlargement of lymph nodes (ELN)		<0.001
Yes	188	
No	933	
Lobe location (LL)		0.773
Left upper lobe	264	
Left lower lobe	167	
Right upper lobe	370	
Right middle lobe	90	
Right upper lobe	230	
Tumor location (TL, zone)		<0.001
Central zone	75	
Middle zone	202	
Peripheral zone	844	
Vascular shadow (VS)		<0.001
Yes	525	
No	596	
Pleural indentation (PI)		<0.001
Yes	638	
No	483	
Lobulated and spiculated sign (LASS)		<0.001
Yes	553	
No	568	
Sex		0.718
Male	406	
Female	715	
Tumor diameter (TD-max)	1.685 ± 0.626 (0.30-3.00)	<0.001
Tumor area (TA-max)	2.573 ± 1.789 (0.18-9.00)	<0.001
The average diameter of the solid components (mediastinal window)	0.727 ± 0.756 (0.00-2.75)	<0.001
The average diameter of the solid components (lung window)	0.869 ± 0.849 (0.00-3.00)	<0.001
The solid component area (SCA, mediastinal window)	1.072 ± 1.527 (0.00-7.56)	<0.001
The solid component area (SCA, lung window)	1.446 ± 1.877 (0.00-9.00)	<0.001
CTR (mediastinal window)	0.315 ± 0.349 (0.00-1.00)	<0.001
CTR (lung window)	0.448 ± 0.451 (0.00-1.00)	<0.001
Age	56.820 ± 9.976 (23.00-84.00)	0.267
CEA	2.724 ± 4.996 (0.12-85.00)	<0.001
NSE	19.016 ± 7.462 (1.35-58.20)	0.079
PaO_2_	89.327 ± 9.836 (42.30-134.00)	0.741
PaCO_2_	40.862 ± 3.551 (22.50-52.80)	0.022

**Table 2:** AUC results (*n* = 1121).

	AUC	95% CI
Tumor diameter (max)	0.797	0.752-0.841
Tumor area (max)	0.801	0.756-0.846
The average diameter of the solid components (mediastinal window)	0.895	0.868-0.923
The average diameter of the solid components (lung window)	0.889	0.861-0.917
The solid component area (mediastinal window)	0.896	0.869-0.923
The solid component area (lung window)	0.888	0.860-0.916
CTR (mediastinal window)	0.873	0.842-0.905
CTR (lung window)	0.824	0.791-0.856

**Table 3:** Test results and evaluation indexes of the RFC model (*n* = 173).

	Result
True-positive (TP) cases	18
True-negative (TN) cases	151
False-positive (FP) cases	2
False-negative (FN) cases	2
Accuracy	0.9769
Sensitivity = recall	0.9000
Specificity	0.9869
Precision	0.9000
95% CI	0.9419-0.9937
*P* value	<0.001
F1	0.9000

**Table 4:** Prediction probability of the test set (*n* = 173).

	Negative probability	Positive probability	Prediction result
1	0.880	0.120	FN
2	0.864	0.136	FN
3	0.994	0.006	TN
4	0.998	0.002	TN
5	0.996	0.004	TN
6	0.980	0.020	TN
7	1.000	0.000	TN
8	1.000	0.000	TN
9	1.000	0.000	TN
10	1.000	0.000	TN
11	1.000	0.000	TN
12	0.932	0.068	TN
13	1.000	0.000	TN
14	1.000	0.000	TN
15	1.000	0.000	TN
16	1.000	0.000	TN
17	1.000	0.000	TN
18	1.000	0.000	TN
19	1.000	0.000	TN
20	0.998	0.002	TN
21	1.000	0.000	TN
22	0.708	0.292	TP
23	0.720	0.280	TP
24	0.476	0.524	TP
25	0.598	0.402	TP
26	0.634	0.366	TP
27	0.646	0.354	TP
28	0.508	0.492	TP
29	0.662	0.338	TP
30	0.546	0.454	TP
31	0.614	0.386	TP
32	0.578	0.422	TP
33	0.702	0.298	TP
34	0.556	0.444	TP
35	0.728	0.272	TP
36	0.712	0.288	TP
37	0.664	0.336	TP
38	0.674	0.326	TP
39	0.730	0.270	TP
40	0.998	0.002	TN
41	1.000	0.000	TN
42	1.000	0.000	TN
43	1.000	0.000	TN
44	0.988	0.012	TN
45	1.000	0.000	TN
46	1.000	0.000	TN
47	0.808	0.192	TN
48	0.970	0.030	TN
49	0.992	0.008	TN
50	0.982	0.018	TN
51	0.982	0.018	TN
52	1.000	0.000	TN
53	0.976	0.024	TN
54	1.000	0.000	TN
55	0.970	0.030	TN
56	1.000	0.000	TN
57	1.000	0.000	TN
58	0.992	0.008	TN
59	0.996	0.004	TN
60	0.988	0.012	TN
61	0.996	0.004	TN
62	0.846	0.154	TN
63	1.000	0.000	TN
64	1.000	0.000	TN
65	1.000	0.000	TN
66	0.926	0.074	TN
67	0.994	0.006	TN
68	0.960	0.040	TN
69	0.982	0.018	TN
70	1.000	0.000	TN
71	0.982	0.018	TN
72	1.000	0.000	TN
73	1.000	0.000	TN
74	1.000	0.000	TN
75	0.992	0.008	TN
76	1.000	0.000	TN
77	1.000	0.000	TN
78	0.950	0.05	TN
79	0.898	0.102	TN
80	0.988	0.012	TN
81	1.000	0.000	TN
82	0.998	0.002	TN
83	0.916	0.084	TN
84	1.000	0.000	TN
85	1.000	0.000	TN
86	0.982	0.018	TN
87	0.998	0.002	TN
88	1.000	0.000	TN
89	1.000	0.000	TN
90	1.000	0.000	TN
91	1.000	0.000	TN
92	1.000	0.000	TN
93	0.928	0.072	TN
94	1.000	0.000	TN
95	0.992	0.008	TN
96	1.000	0.000	TN
97	0.976	0.024	TN
98	0.834	0.166	TN
99	1.000	0.000	TN
100	1.000	0.000	TN
101	0.996	0.004	TN
102	0.936	0.064	TN
103	0.952	0.048	TN
104	1.000	0.000	TN
105	0.970	0.030	TN
106	0.992	0.008	TN
107	1.000	0.000	TN
108	0.996	0.004	TN
109	0.998	0.002	TN
110	0.984	0.016	TN
111	1.000	0.000	TN
112	0.996	0.004	TN
113	0.994	0.006	TN
114	1.000	0.000	TN
115	0.998	0.002	TN
116	0.904	0.096	TN
117	0.958	0.042	TN
118	0.998	0.002	TN
119	1.000	0.000	TN
120	1.000	0.000	TN
121	0.852	0.148	TN
122	1.000	0.000	TN
123	1.000	0.000	TN
124	0.860	0.140	TN
125	1.000	0.000	TN
126	1.000	0.000	TN
127	0.950	0.050	TN
128	1.000	0.000	TN
129	0.996	0.004	TN
130	0.824	0.176	TN
131	0.962	0.038	TN
132	0.810	0.190	TN
133	0.984	0.016	TN
134	0.926	0.074	TN
135	0.896	0.104	TN
136	0.960	0.040	TN
137	1.000	0.000	TN
138	0.996	0.004	TN
139	0.956	0.044	TN
140	1.000	0.000	TN
141	0.920	0.080	TN
142	0.794	0.206	FP
143	0.994	0.006	TN
144	0.996	0.004	TN
145	1.000	0.000	TN
146	1.000	0.000	TN
147	0.936	0.064	TN
148	1.000	0.000	TN
149	0.998	0.002	TN
150	0.748	0.252	FP
151	0.996	0.004	TN
152	0.920	0.080	TN
153	1.000	0.000	TN
154	1.000	0.000	TN
155	0.996	0.004	TN
156	1.000	0.000	TN
157	0.996	0.004	TN
158	1.000	0.000	TN
159	1.000	0.000	TN
160	1.000	0.000	TN
161	0.998	0.002	TN
162	1.000	0.000	TN
163	0.996	0.004	TN
164	0.974	0.026	TN
165	1.000	0.000	TN
166	0.904	0.096	TN
167	1.000	0.000	TN
168	1.000	0.000	TN
169	1.000	0.000	TN
170	0.990	0.010	TN
171	1.000	0.000	TN
172	1.000	0.000	TN
173	1.000	0.000	TN

## Data Availability

Research data supporting this publication are available. If you need this database, please contact us (Email: 2019122062@cmu.edu.cn).

## Abbreviations

CTR: Consolidation tumor ratio
CEA: Carcinoembryonic antigen
CT: Computed tomography
ELN: Enlargement of lymph nodes
LA: Lung adenocarcinoma
LASS: Lobulated and spiculated sign
LL: Lobe location
LNM: Lymph node metastasis
ML: Machine learning
NSCLC: Non-small-cell lung cancer
NSE: Neuron-specific enolase
OLM: Occult lymph node metastasis
PI: Pleural indentation
RFC: Random forest classifier
SCA: Solid component area
TA: Tumor area
TD: Tumor diameter
TL: Tumor location
VATS: Video-assisted thoracic surgery
VS: Vascular shadow.
